# Early Pregnancy Serum Metabolite Profiles Associated with Hypertensive Disorders of Pregnancy in African American Women: A Pilot Study

**DOI:** 10.1155/2020/1515321

**Published:** 2020-02-19

**Authors:** Erin P. Ferranti, Jennifer K. Frediani, Rebecca Mitchell, Jolyn Fernandes, Shuzhao Li, Dean P. Jones, Elizabeth Corwin, Anne L. Dunlop

**Affiliations:** ^1^Nell Hodgson Woodruff School of Nursing, Emory University, 1520 Clifton Road, Rm 436, Atlanta, GA 30322, USA; ^2^Nell Hodgson Woodruff School of Nursing, Department of Computer Science, Emory University, 1520 Clifton Road, Rm 436, Atlanta, GA 30322, USA; ^3^Department of Medicine, Emory University, 1520 Clifton Road, Rm 436, Atlanta, GA 30322, USA

## Abstract

Hypertensive disorders of pregnancy (HDP) are the most common cardiometabolic complications of pregnancy, affecting nearly 10% of US pregnancies and contributing substantially to maternal and infant morbidity and mortality. In the US, women of African American race are at increased risk for HDP. Early biomarkers that reliably identify women at risk for HDP remain elusive, yet are essential for the early identification and targeting of interventions to improve maternal and infant outcomes. We employed high-resolution metabolomics (HRM) to identify metabolites and metabolic pathways that were altered in early (8-14 weeks) gestation serum samples of pregnant African American women who developed HDP after 20 weeks' gestation (*n* = 20)—either preeclampsia (PE; *n* = 11) or gestational hypertension (gHTN; *n* = 9)—compared to those who delivered full term without complications (*n* = 80). We found four metabolic pathways that were significantly (*p* < 0.05) altered in women who developed PE and five pathways that were significantly (*p* < 0.05) altered in women who developed gHTN compared to women who delivered full term without complications. We also found that four specific metabolites (*p* < 0.05) were distinctly upregulated (retinoate, kynurenine) or downregulated (*SN*-glycero-3-phosphocholine, 2′4′-dihydroxyacetophenone) in women who developed PE compared to gHTN. These findings support that there are systemic metabolic disruptions that are detectable in early pregnancy (8-14 weeks of gestation) among pregnant African American women who develop PE and gHTN. Furthermore, the early pregnancy metabolic disruptions associated with PE and gHTN are distinct, implying they are unique entities rather than conditions along a spectrum of the same disease process despite the common clinical feature of high blood pressure.

## 1. Introduction

Hypertensive disorders of pregnancy (HDP) include gestational hypertension (gHTN), preeclampsia (PE) that is de novo or superimposed on preexisting chronic hypertension, and preexisting chronic hypertension [1]. By conservative estimates, HDP are responsible for 76,000 maternal and 500,000 infant deaths globally each year. Together they are the most common cardiometabolic complications of pregnancy, affecting nearly 10% of US pregnancies [2] and have been increasing in recent years [3]. In the US, women of African American race are at the highest risk for developing HDP, and their overall pregnancy-related mortality ratio is 3.4 times higher relative to white women [4]. Risk factors for gHTN include obesity, maternal age less than 20 or more than 40 years, and family history. Risk factors for PE are less understood but include both nulliparity and grand multiparity, history of PE or chronic hypertension, diabetes, renal disease, and obesity [5, 6]. Despite the tremendous morbidity and mortality burden of HDP, the etiology and pathogenesis remain elusive, thereby limiting the development of specific preventive and treatment strategies. There is a need to identify early pregnancy clinical biomarkers of risk to enhance the timely targeting of treatment.

Systems biology and high-throughput omics technologies offer great promises for understanding molecular mechanisms of disease risk and pathogenesis. In particular, high-resolution metabolomics (HRM) provides the most comprehensive measurement of small molecules (metabolites) in biological samples and can identify metabolic pathways that are activated or deactivated in health or disease [7]. HRM uses liquid chromatography (LC) or gas chromatography (GC) with high-resolution mass spectrometry (HRMS) and advanced data extraction algorithms to measure a broad spectrum of chemicals in biologic samples [8]. The approach complements targeted metabolomics through the use of computational methods for metabolite and pathway analysis [9]. HRM is a promising tool for uncovering metabolites and metabolic pathways that may be perturbed early in the trajectory of metabolic dysfunction and may serve to identify early onset or those at risk for the condition.

A few recent studies have applied HRM to identify metabolites and metabolic pathways that are altered in PE [10–14], and to our knowledge, only one study also examined gHTN [15]. African American (AA) women are underrepresented in these published studies. The purpose of the current study was to expand this body of inquiry and employ HRM on early (8-14 weeks) gestation serum samples to identify metabolites and metabolic pathways that were altered among AA women who developed HDP (PE and/or gHTN) compared to those who delivered full term without complications.

## 2. Materials and Methods

### 2.1. Study Design and Population

This nested case-control study was undertaken on a subset of women who were enrolled in the Emory University African American Vaginal, Oral, and Gut Microbiome in Pregnancy Cohort Study, which is described in detail elsewhere [16]. Briefly, pregnant women aged 18-40 years presenting with a singleton pregnancy at 8-14 weeks of gestation for prenatal care to clinics affiliated with Grady Memorial Hospital, a publicly-funded hospital, or Emory Midtown Hospital, a private hospital, who self-identified as AA are invited to participate in this ongoing cohort study. Exclusion criteria for study entry include the presence of any chronic medical condition, including chronic hypertension. Those who meet the criteria and provide informed consent undergo data collection at the time of enrollment (8-14 weeks of gestation). The women were enrolled between June 2014 and August 2015 and had venous blood collected at 8-14 weeks of gestation; these blood samples were then analyzed in a single batch to eliminate any opportunity for batch effects not accounted for during analytic processing. The women selected as cases were those who had a diagnosis of either PE or gHTN during the pregnancy (*n* = 20), while controls were those whose pregnancy ended in a full-term birth (between 39-0/7 and 40-6/7 weeks of gestation) without cardiometabolic complications of pregnancy (*n* = 80). Women who experienced gestational diabetes were excluded from all analysis.

### 2.2. Data Collection

Data collection occurred at the enrollment visit (between 8 and 14 weeks of gestation) and via medical record abstraction completed postdelivery to capture the birth and pregnancy outcomes over the course of the pregnancy. Measures collected as part of the larger cohort study that are relevant to this study include the following.

Sociodemographic survey based on maternal self-report and prenatal administrative record review was used to ascertain maternal age, level of education, marital status, and insurance status.

Medical chart abstraction was completed by the research team using a standardized chart abstraction tool to ascertain the following pre- and perinatal characteristics, conditions, and birth outcomes. (1) *Parity* is categorized according to whether the woman had had any prior term or preterm birth. (2) *Prepregnancy body mass index* (*BMI*) is calculated from measured height and weight at the first prenatal visit between 8 and 14 weeks of gestation and categorized according to accepted definitions (obesity ≥ 30 kg/m^2^, overweight 25-29.99 kg/m^2^, healthy weight 18.5-24.99 kg/m^2^, and underweight < 18.5 kg/m^2^). (3) *Gestational age at birth*: all participants received early pregnancy dating by last menstrual period (LMP) and/or early ultrasound, given enrollment criteria. Gestational age at birth was determined from the delivery record using upon the best obstetrical estimate [17], based upon the date of delivery in relation to the estimated date of confinement established by the 8- to 14-week prenatal visit (39 0/7 weeks of gestation through 40 6/7 weeks of gestation). (4) *Cardiometabolic complications of pregnancy*: women were considered to have gestational hypertension (gHTN) if they had new-onset hypertension (defined as systolic blood pressure ≥ 140 mmHg and/or diastolic blood pressure ≥ 90 mmHg at ≥20 weeks of gestation in the absence of proteinuria or new signs of end-organ dysfunction (with blood pressure readings documented on at least two occasions at least four hours apart)) and were considered to have preeclampsia if they met the same blood pressure criteria along with proteinuria or new signs of end-organ dysfunction [18]. Cases of preeclampsia were further categorized as early onset if preeclampsia developed before 34 weeks of gestation and as late onset if preeclampsia developed at or after 34 weeks of gestation [19]. Women were considered to have gestational diabetes if they developed glucose intolerance after 20 weeks of gestation, as diagnosed by an abnormal oral glucose tolerance test [20]. Venous blood draw was completed by a phlebotomist during the enrollment encounter. Serum aliquots from the venous blood were obtained and stored at -80 degrees Celsius until later analysis of serum metabolites.

### 2.3. High-Resolution Serum Metabolomics

HRM was completed using LC-HRMS (liquid chromatography coupled high-resolution mass spectrometry) [21, 22]. Serum samples were prepared and analyzed in batches of 20; each batch included duplicate analysis of pooled human serum for quality control purposes and reference standardization. Prior to analysis, serum aliquots were removed from storage at -80°C and thawed on ice. Each cryotube was vortexed briefly to ensure homogeneity and 50 *μ*L transferred to a clean microfuge tube. Immediately after, serum was treated with 100 *μ*L of ice-cold LC-MS grade acetonitrile (Sigma-Aldrich) containing 2.5 *μ*L of internal standard solution with eight stable isotopic chemicals selected to cover a range of chemical properties. Following addition of acetonitrile, serum was equilibrated for 30 min on ice, upon which precipitated proteins were removed by centrifuge (16.1g at 4°C for 10 min). The resulting supernatant (100 *μ*L) was removed, added to a low-volume autosampler vial, and maintained at 4°C until analysis (<22 h).

Samples were analyzed in triplicate using 10 *μ*L injections and separate HILIC chromatography columns with detection by high-resolution mass spectrometry (Thermo Scientific Dionex Ultimate 3000RSLCnano, Thermo Scientific Orbitrap Fusion Tribrid Mass Spectrometer). During HILIC chromatography, the electrospray ionization (ESI) source is operated in positive ion mode.

The high-resolution mass spectrometer was operated in full scan mode at 120,000 resolution and mass-to-charge ratio (*m*/*z*) range 85-1275. Raw data files were extracted and aligned using apLCMS [23] and xMSanalyzer [24]. Uniquely detected ions consisted of accurate mass *m*/*z*, retention time, and ion abundance, referred to as *m*/*z* features. Prior to data analysis, *m*/*z* features were batch corrected using ComBat [25]. Data from HILIC column with positive ion mode were used for this analysis.

### 2.4. Metabolite Annotation

For the HRM method used, identities of more than 400 metabolites have been confirmed by retention time and MS/MS fragmentation criteria relative to authentic standards (Schymanski et al. [26]) Level 1 criteria. For the workflow used, metabolic features were annotated using xMSannotator in which the confidence scores for annotation are derived from a multistage clustering algorithm [27]. For features selected using linear regression and partial least squares-discriminant analysis (PLS-DA), identification of the metabolites was confirmed by criteria of Schymanski et al. [26], either by Level 1 identification, which involves comparing mass spectrum and coelution relative to authentic standards, or by Level 2 identification, which involves comparison to METLIN spectral database (http://metlin.scripps.edu/index.php). Lower confidence annotations designated as Level 3-5 identification by Schymanski et al. [26] were made using HMDB (Human Metabolome Database, http://www.hmdb.ca/) [28] and KEGG (Kyoto Encyclopedia of Genes and Genomes, http://www.genome.jp/kegg/) [29]. Additional manual search was done using METLIN at 5 ppm tolerance [30]. Only metabolites corresponding to Level 1 identification are reported in this manuscript.

### 2.5. Bioinformatics and Statistics

Descriptive statistics were used to evaluate participant characteristics, using chi-square or Fisher's exact test as appropriate for categorical variables and Student's *t*-test for continuous variables. A one-way analysis of variance (ANOVA) was calculated on demographic and clinical variables to determine differences between the three groups. For any test that failed homogeneity of variance, we used the Welch's robust test of equality.

Metabolomics data were filtered to remove features not present in at least 80% of one group or >80% of all samples. After filtering, missing values were imputed by one-half of the lowest signal detected for that feature across all samples [31]. Data were then log_2_ transformed and quantile normalized [32, 33]. Generalized linear regression methods were used to compare groups (PE vs. healthy controls, gHTN vs. healthy controls, and PE vs. gHTN) and control for covariates (age, prior term pregnancy, prior preterm pregnancy, and first prenatal BMI). Each *m*/*z* feature is used as the independent variable while class (comparisons described above) and other covariates act as dependent variables. Multiple hypothesis correction was performed using the Benjamini-Hochberg false discovery rate (FDR) correction method with threshold *q* value of 0.20 [34]. Because the goal of this study was to explore metabolic differences among women with and without hypertensive disorders in pregnancy for future hypothesis testing, less stringent methods were used (raw *p* values vs. FDR threshold) in all visualization methods. Previously published studies have shown that FDR correction results in type II statistical error while protecting for type I statistical error [35]. Pathway enrichment analysis using features significant at raw *p* value provides a 2-step approach which protects against both type I and type II errors [8]. In order to explore the direct comparison between hypertensive disorders (PE vs. gHTN), we used partial least squares-discriminant analysis (PLS-DA) and used a Variable Importance for Projection (VIP) > 2 for further annotation. Type 1 (-log_10_*p* vs. *m*/*z*) and Type 2 (-log_10_*p* vs. retention time) Manhattan plots were used to visualize the pattern of differential expression across all features with respect to molecular mass and chemical properties, respectively. Discriminatory features were analyzed through an unsupervised multivariate approach using principal component analysis (PCA) to visualize the participant samples. All analysis was performed using several R packages using an automated workflow package (xmsPANDA version 1.0.7.4) and R version 3.4.3.

Mummichog v2.0 was used to perform pathway enrichment analysis using *m*/*z* features that were significant at *p* < 0.05 and had VIP > 1 [36]. Mummichog was designed to perform pathway and network analysis for untargeted metabolomics. The software compares the enrichment pattern of the significant metabolite subsets with null distribution on known metabolic reactions and pathways, thereby allowing prioritization of pathways for further evaluation [8].

## 3. Results

### 3.1. Study Sample Characteristics

The study sample included 100 participants: 20 who developed HDP (11 PE, 9 gHTN) and 80 who had uncomplicated, full-term pregnancies. Of the 11 cases of PE, a single case was early onset (with onset occurring at 33 weeks and 5 days of gestation), while the remainder onset at or after 34 weeks of gestation. Demographic and clinical characteristics of participating women according to case or control status are given in Table 1; with exception to parity, these characteristics did not vary across the three groups. Nulliparity is a known risk factor for HDP and was controlled for in the analyses.

To determine if there were early pregnancy metabolic differences between women who developed HDP (*n* = 20) and controls (*n* = 80), HRM was performed and provided 16,481 *m*/*z* features. In addition, women who developed particular subtypes of HDP, including PE (*n* = 11) and gHTN (*n* = 9), were compared to controls (*n* = 80) and to each other to determine potential discriminatory features, as described below. The number of features considered in each comparison differs as the samples included in a given comparison differ according to the clinical outcome groups being compared. No metabolites were significant by FDR *q* value of 0.20.

#### 3.1.1. Preeclampsia vs. Healthy Controls

**Figure 1:**
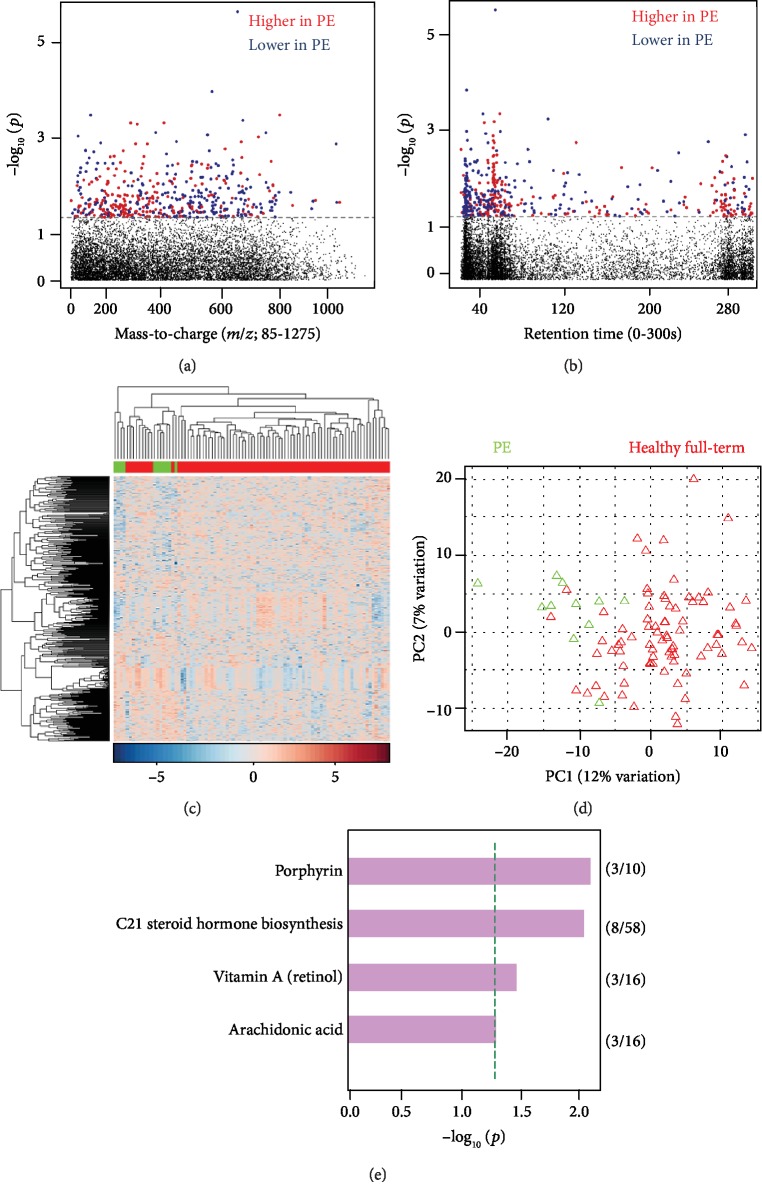
Metabolic profiles of women with PE versus healthy controls. (a) Type 1 Manhattan plot, -log_10_*p* vs. mass-to-charge ratio. 470 *m*/*z* features were found significant at *p* value 0.05. No metabolites were significant by false discovery rate (FDR) *q* value 0.20. Red dots represent those features upregulated in preeclampsia (PE), and the blue dots represent features that were downregulated in PE; the dashed line represents significance cut-off of *p* value < 0.05. (b) Type 2 Manhattan plot, -log_10_*p* vs. retention time, the majority of features had retention time below 2 minutes; the dashed line represents significance cut-off of *p* value < 0.05. (c) 2-way hierarchical cluster analysis, PE is represented in green and healthy full term in red across the *x*-axis; significant features are clustered on the *y*-axis. (d) Principal component analysis. (e) Mummichog-enriched pathways at *p* value < 0.05 represented by the green dotted line.

After preprocessing, 9326 features remained for analysis. Using a multivariate approach, controlling for parity, maternal age, and first prenatal body mass index (BMI), 470 significant features (raw *p* value < 0.05) were selected as discriminatory between PE (*n* = 11) and healthy, full-term controls (*n* = 80) (Figures 1(a) and 1(b)). Manhattan plots show discriminatory features with a broad range of both mass-to-charge ratio (Figure 1(a)) and retention time (Figure 1(b)). Two-way hierarchical cluster analysis aided in visualization of correlations between biological samples and metabolic features, showing distinct clustering for cases vs. controls (Figure 1(c)). Principal component analysis (PCA) was performed using the discriminatory features to visualize the differences between biological samples (Figure 1(d)). Pathway enrichment analysis using Mummichog showed significant enrichment (*p* value < 0.05) of pathways that have been indicated in blood pressure regulation including porphyrin metabolism, steroid hormone biosynthesis, and vitamin A and arachidonic acid metabolism (Figure 1(e)).

#### 3.1.2. Gestational Hypertension vs. Healthy Controls

**Figure 2:**
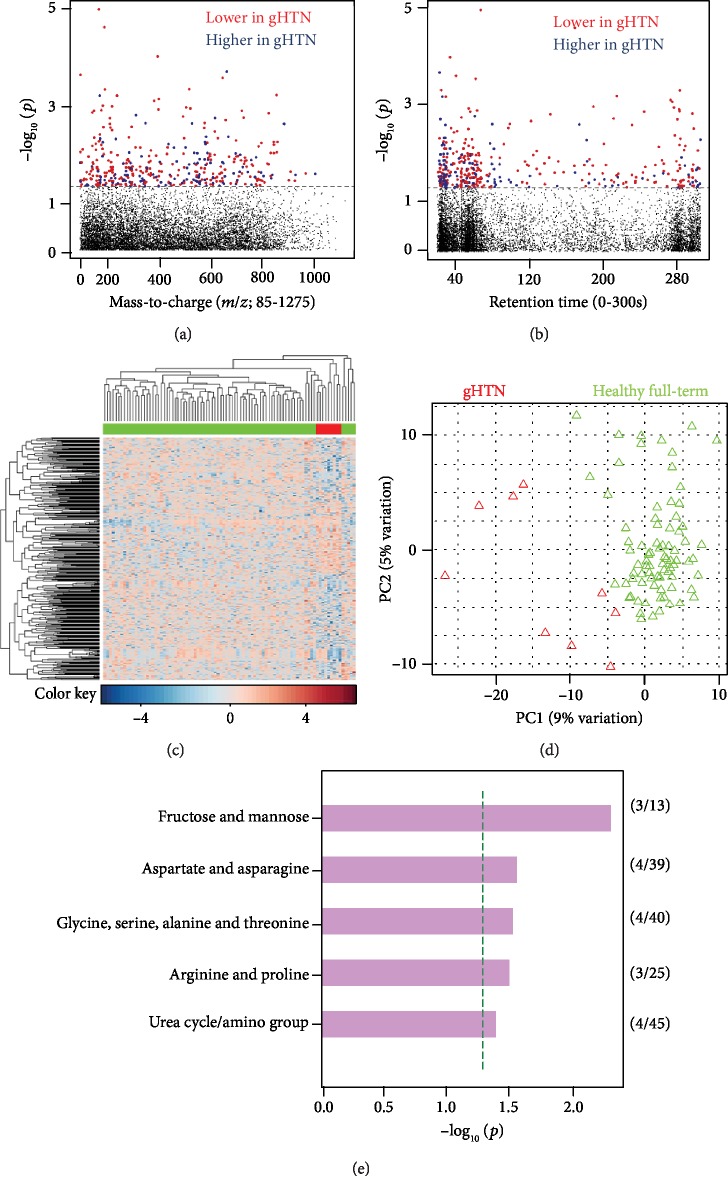
Metabolic profiles of women with gestational hypertension versus healthy controls. (a) Type 1 Manhattan plot, -log_10_*p* vs. mass-to-charge ratio. 388 *m*/*z* features were found significant at *p* value 0.05. No metabolites were significant by false discovery rate (FDR) *q* value 0.20. Red dots represent those features downregulated in gestational hypertension (gHTN), and the blue dots represent features that were downregulated in healthy full-term women; the dashed line represents significance cut-off of *p* value < 0.05. (b) Type 2 Manhattan plot, -log_10_*p* vs. retention time. Majority of features had retention time below 2 minutes; the dashed line represents significance cut-off of *p* value < 0.05. (c) 2-way hierarchical cluster analysis. gHTN is represented in red and healthy full term in green across the *x*-axis; significant features are clustered on the *y*-axis; there is a clean separation seen between biological samples. (d) Principal component analysis. (e) Mummichog-enriched pathways at *p* value < 0.05 represented by the green dotted line.

After preprocessing, 9270 features remained for analysis. Using a multivariate approach, 388 significant features (raw *p* value < 0.05) were selected as discriminatory between gHTN (*n* = 9) and healthy control (*n* = 80) (Figures 2(a) and 2(b)). Manhattan plots show discriminatory features with a broad range of both mass-to-charge ratio (Figure 2(a)) and retention time (Figure 2(b)). Two-way hierarchical cluster analysis aided in visualization of correlations between biological samples and metabolic features and showed distinct clustering among case vs. control (Figure 2(c)). Principal component analysis (PCA) was performed using the discriminatory features to visualize the differences between biological samples (Figure 2(d)). Pathway enrichment analysis using Mummichog showed significant enrichment (*p* value < 0.05) of pathways related to fructose and mannose metabolism; amino acid metabolism involving aspartate, asparagine, glycine, serine, alanine, threonine, arginine, and proline; and urea cycle metabolism (Figure 2(e)).

#### 3.1.3. Preeclampsia vs. Gestational Hypertension

**Figure 3:**
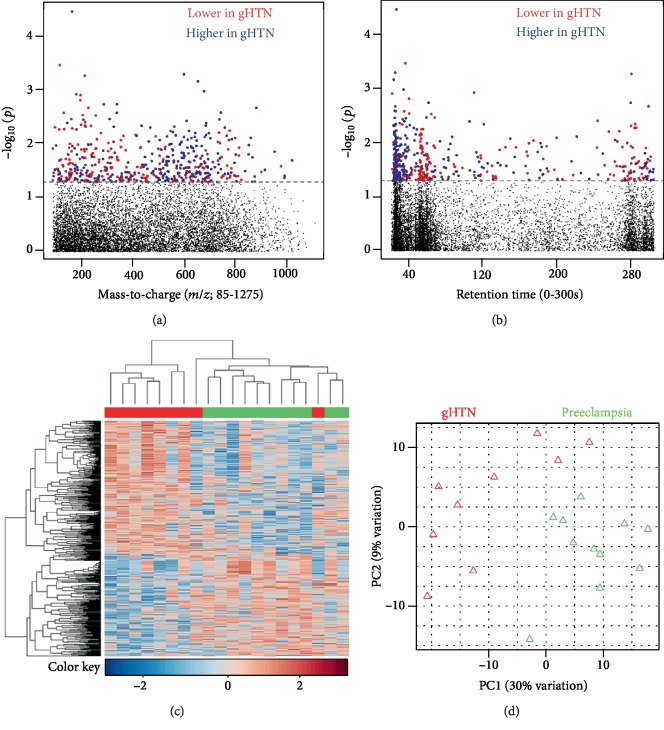
Preeclampsia vs. gestational hypertension. (a) Type 1 Manhattan plot, -log_10_*p* vs. mass-to-charge ratio. 486 *m*/*z* features were found significant at *p* value 0.05. No metabolites were significant by false discovery rate (FDR) *q* value 0.20. Red dots represent those features downregulated in gestational hypertension (gHTN), and the blue dots represent features that were downregulated in PE. (b) Type 2 Manhattan plot, -log_10_*p* vs. retention time. (c) 2-way hierarchical cluster analysis. gHTN is represented in red and preeclampsia in green across the *x*-axis; significant features are clustered on the *y*-axis; there is clear separation between the two groups of women. (d) Principal component analysis.

After preprocessing, 8954 features remained for analysis. Using a multivariate approach, 486 significant features (raw *p* value < 0.05) were selected as discriminatory between PE (*n* = 11) and gHTN (*n* = 9) (Figures 3(a) and 3(b)). Manhattan plots show discriminatory features with a broad range of both mass-to-charge ratio (Figure 3(a)) and retention time (Figure 3(b)). Two-way hierarchical cluster analysis aided in visualization of correlations between biological samples and metabolic features and showed distinct clustering among gHTN (*n* = 9) vs. PE (*n* = 11) (Figure 3(c)). Principal component analysis (PCA) was performed using the discriminatory features to visualize the differences between biological samples (Figure 3(d)). Pathway enrichment analysis using Mummichog showed significant enrichment (*p* value < 0.05) of the vitamin A metabolism pathway.

**Figure 4:**
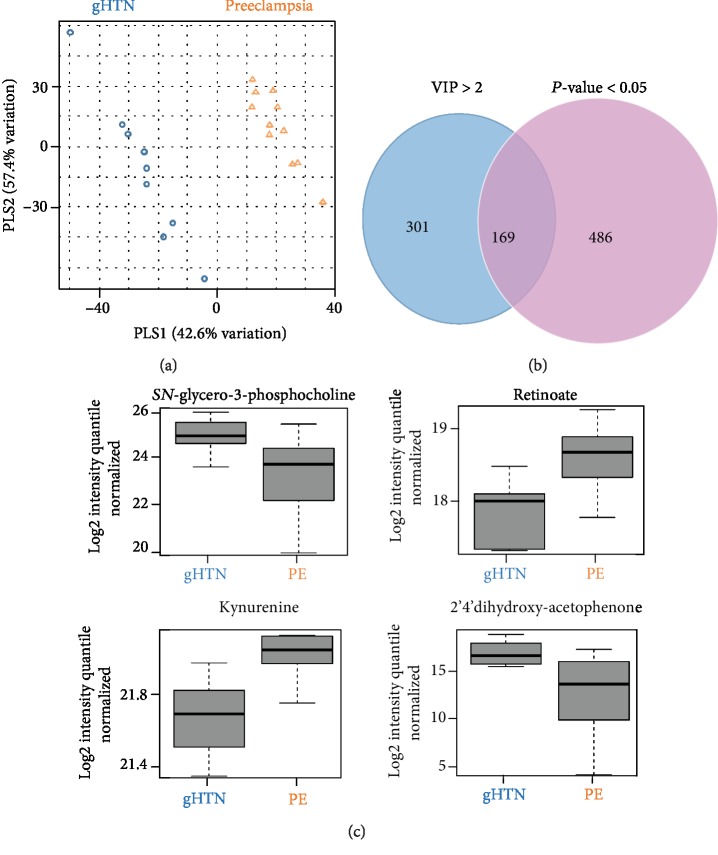
Gestational hypertension vs. preeclampsia. (a) Partial least squares-discriminant analysis (PLS-DA). This supervised discriminatory analysis shows preeclampsia (PE) in orange triangles and gestational hypertension (gHTN) in blue circles. (b) Venn diagram showing 169 overlapping significant features between linear regression (*p* value < 0.05) and PLS-DA (VIP > 2). (c) Box and whisker plots for 4 significant verified features (from left to right) *SN*-glycero-3-phosphocholine (*m*/*z* 258.1094; RT 76 s), retinoate (*m*/*z* 301.2174; RT 28 s), L-kynurenine (*m*/*z* 209.0922; RT 40 s), and 2′4′-dihydroxyacetophenone (*m*/*z* 153.0577; RT 42 s).

To select the features most likely to discriminate between women who developed PE (*n* = 11) and those with gHTN (*n* = 9), Variable Importance for Projection (VIP) scores were obtained from a partial least squares-discriminant analysis (PLS-DA) model. There were 169 features significant both by *p* value < 0.05 and VIP > 2 (Figures 4(a) and 4(b)). Of those, four significant features were verified and designated by Schymanski Level 1 criteria, including *SN*-glycero-3-phosphocholine, retinoate, kynurenine, and 2′4′-dihydroxyacetophenone (Figure 4(c)).

A summary of the metabolic pathways showing significant enrichment (*p* value < 0.05) based on Mummichog pathway enrichment analysis for the intergroup comparisons that were made is shown in Table 2. When examining results for PE (*n* = 9) vs. healthy controls (*n* = 80) and gHTN (*n* = 9) vs. healthy controls (*n* = 80), it is notable that among the pathways for which there was significant enrichment there were no overlapping pathways. When examining results for PE (*n* = 11) vs. healthy controls (*n* = 80) and PE (*n* = 11) vs. gHTN (*n* = 9), the vitamin A metabolic pathway was significantly enriched in both comparisons.

## 4. Discussion

In this high-resolution metabolomics study, we identified key serum metabolites and metabolic pathways that were altered in early pregnancy (8-14 weeks of gestation) in AA women who developed HDP (*n* = 20) compared to those who delivered full term without complications (*n* = 80). To our knowledge, this is the first metabolome study of HDP to focus solely on pregnant African American women, the population of women most severely affected by HDP [37]. We examined two HDP conditions—both gHTN and PE—and identified distinct metabolite and metabolic pathway differences between the two. Furthermore, when we compared gHTN to PE with discriminatory analyses, we found greater variation in the conditions examined separately, when compared to controls, than when combined into a single category. This finding together with the four distinct metabolites that were differentiated when we compared gHTN and PE suggests that gHTN and PE are distinct entities with unique mechanistic pathways and not necessarily a spectrum in severity of the same disease process, despite the common clinical feature of high blood pressure [5].

There were four pathways that were significantly enriched in women who developed PE (*n* = 11) compared to women with healthy, full-term pregnancies (*n* = 80) in our study. One of those pathways was related to porphyrin metabolism. Alterations in porphyrin metabolism have been reported to be associated with porphyria, a group of enzymatic deficiency disorders of the heme biosynthetic pathway [38]. Porphyrias are classified as either erythropoietic or hepatic based upon where the overproduction of porphyrins occurs or classified based on symptomology. The individual metabolites that were altered in our sample of women who developed PE were those associated with the liver. Porphyrias are also classified as either chronic or acute. Chronic or cutaneous porphyrias affect the skin, while the acute porphyrias affect the nervous system. One of the acute forms is acute intermittent porphyria (AIP). While only 10-15% of gene carriers demonstrate the clinical syndrome, symptoms are often unrecognized or misdiagnosed and can become exacerbated during pregnancy. Though not well-studied, AIP has been linked with adverse pregnancy outcomes, including pregnancy-induced hypertension [39], while altered porphyrin metabolism has been linked with PE in individual case studies [40, 41]. It is unknown if any of our participants had AIP, since this condition is not routinely screened in clinical care.

A second pathway that was significantly altered in PE was steroid hormone biosynthesis. Clinically, women with PE will often present with high circulating testosterone and reduced estradiol production and are considered to experience endocrine disorders [42, 43]. This has been demonstrated in pregnant AA women as well [44]. Given that the estradiol metabolite was upregulated in our sample, our findings conflict with these previous findings. However, another very recent study has revealed that a high level of testosterone in PE patients could lead to a marked suppression in estrogen production via targeting of the specific estrogen receptors in the placenta [45]. The placenta is an important source of steroid hormone production throughout gestation, and placental deficiencies are widely recognized as a principal pathological component of PE [46].

Another pathway of steroid hormone biosynthesis involves glucocorticoids. Hyperactivity of the hypothalamic pituitary adrenal (HPA) axis and hypercortisolism is a normal physiologic response in healthy pregnancies [47]. Both PE and gHTN have been characterized by lower cortisol levels as compared to normotensive pregnant women, suggesting that there may be increased cortisol metabolism in these conditions [48, 49]. Our findings support this decreased level with a downregulation of the tetrahydrocorticosterone metabolite.

The retinol metabolism pathway was altered in women with PE, with all three metabolites in this pathway downregulated. Retinol is a key vitamin in pregnancy with its role in cell differentiation and the growth and development of the embryo [50]. Retinol binding protein 4 (RBP4) is the adipokine that delivers retinol from the liver to the peripheral tissue. Although retinol metabolism has not been previously identified as a significant factor in PE, there are numerous studies that have examined the role of RBP4, and to date, the findings are mixed with some studies finding upregulation, others finding downregulation, and some finding no differences [51–54].

Finally, in women who later developed PE (*n* = 11) in our study, the fourth metabolic pathway to show alterations was that of arachidonic acid metabolism. Three metabolites within the arachidonic acid metabolism were downregulated in women who later developed PE and included leukotriene A4, 13,14-dihydroxy-retinol, and semialdehyde. The association with arachidonic acid metabolism and PE has been described before [55] and appears to be related to the lipid peroxidation and oxidative stress [56], although the findings are mixed and inconclusive [57]. However, a more recent study published has also identified that placental metabolites of arachidonic acid are altered in PE and may contribute to the increases in placental vascular resistance, alterations in uterine hemodynamics, and dysregulation of placental vascular remodeling seen in women with PE [58].

As compared to PE, gHTN has not been well studied using metabolomics technologies. In our study, women with gHTN had five metabolic pathways that were significantly enriched with four involving amino acid metabolism or catabolism and one involving fructose metabolism. Diets high in fructose consumption have been linked with cardiometabolic disease [59], and recent animal studies have shown an association between maternal fructose consumption and the incidence of hypertension in their offspring [60].

Alterations in amino acid metabolism have been linked with hypertension in nonpregnant populations [61], and specific combined amino acid pathways (such as forglycine/serine/alanine/threonine) have also been found to be significantly altered with metabolic syndrome [62]. In pregnancy, many of the amino acid pathways we found to be associated with gHTN have been associated with PE in other studies, including arginine, alanine, serine, glycine, and asparagine [11, 14, 63]. Of particular significance is our finding related to altered metabolism with arginine given its role as the immediate precursor of nitric oxide, which is involved in vasodilation and blood pressure regulation [63]. In the only other published study that specifically used metabolomics technologies to examine gHTN in pregnancy, the development of gHTN was associated with upregulated triglycerides and downregulated high-density lipoprotein, lactate, N-acetyl glycoproteins, phosphatidylcholine, and glucose in early gestation (11-13 weeks) [15].

When we combined gHTN and PE into one group of HDP and compared to women with healthy, full-term pregnancies (data included in Supplemental file ([Supplementary-material supplementary-material-1])), the only pathway to remain significant was that of vitamin A, which we also found in the PE group alone and as a specific metabolite among the PE group (*n* = 11) when compared to women with gHTN (*n* = 9). The significance of this metabolite in relation to PE was described earlier. Additionally, when we compared gHTN (*n* = 9) to PE (*n* = 11), we found three additional distinct metabolites that were either upregulated or downregulated in either condition. *SN*-glycero-3-phosphocholine is a phospholipid that serves a precursor to choline biosynthesis and an intermediate in the metabolism of phosphatidylcholine. Choline metabolites have been associated with unfavorable cardiometabolic risk factors, while phosphatidylcholine has been associated with a lower odds of hypertension [64]. L-Kynurenine is a metabolite derived from the amino acid L-tryptophan and used in the production of niacin. It has also been found to be important for arterial vessel relaxation and control of blood pressure [65, 66]. The final metabolite which was lower in the PE group as compared to the gHTN group was *2*′*4*′*-dihydroxyacetophenone.* This is a chemical compound used as a flavoring ingredient in food (http://www.hmdb.ca/) and has not been investigated with its role in PE, hypertension, or any other health outcome.

In summary, our findings highlight some metabolic functions and pathways that have been described before in HDP and also identify new and novel metabolites and pathways that are unique to PE and gHTN, specifically in a population of AA women. Our study also provides evidence that the metabolic signatures of these two conditions are unique, with little or no overlap apparent. As only metabolites corresponding to Level 1 identification, meaning those metabolites whose identity is confirmed by retention time and MS/MS fragmentation criteria relative to authentic standards [27], were reported in this manuscript, we have confidence in the identity of the metabolites for which intergroup differences were found.

Although our study has important strengths as highlighted previously, some limitations are worth noting. First, the sample size of this study is small, though comparable to other pilot metabolomics studies, while representing a unique focus solely on African American women, a population with the most disparate rates and outcomes related to hypertensive disorders of pregnancy [12, 15]. Because of the small sample size, however, the results of this study should be interpreted as exploratory; dedicated studies on a larger sample size are needed to confirm the findings from this study. Furthermore, the small sample size, with only a single case of PE that was early onset and the remaining 10 cases being late onset, limited our ability to evaluate differences between early- vs. late-onset disease. Using nuclear magnetic resonance spectrometry, a platform substantially different from the LC-HRMS (liquid chromatography coupled high-resolution mass spectrometry) platform used in this study, Bahado-Singh et al. did find significant differences in some metabolites in the first-trimester serum samples obtained from those who later developed late-onset vs. early-onset PE [11]. As these analyses represent an initial, pilot study designed to be hypothesis generating, our findings will help inform our next analyses in a larger sample of women with PE, including more cases of both early-onset and late-onset PE. Second, our window of data collection was limited to women who were clinically verified to be between 11 and 14 weeks of gestation, but that range in gestational age could contribute to variability in pregnancy-specific metabolites and subclinical HDP disease progression—both conditions that have received little study to date, so it is unknown how they might confound our findings.

The use of metabolomics for the early identification of metabolites associated with PE is of growing interest [67]. While most studies have identified particular metabolites as significantly associated with or predictive of the later development of PE, the field is still young and it is challenging to compare findings due to variability in study design, biospecimen type, gestational age and disease severity at the time of sampling, and methods of bioinformatics analyses. Our analyses in particular included identifying metabolic pathways using Mummichog [36], which has not previously been reported in metabolomics studies of HDP.

Metabolomics is a powerful approach for identifying the underlying mechanisms, metabolites, and metabolic pathways associated with the spectrum of HDP. Our study adds to the growing body of literature using metabolomics technologies to identify early metabolites and metabolic pathways that may signal important mechanisms early in the trajectory of HDP development, specifically in both gHTN and PE. The ability to accurately identify women at risk of developing HDP is essential to enable early initiation of interventions to improve maternal and infant outcomes. As the population most severely impacted by HDP, African American women must continue to be well represented in HDP metabolomics studies. Continued progress in this field of study should enable greater understanding of the mechanisms and etiology of HDP, which will inform future preventive and treatment efforts.

## Figures and Tables

**Table 1:** Demographics and clinical characteristics of participants.

	Full-term, healthy controls *n* = 80	Gestational hypertension *n* = 9	Preeclampsia *n* = 11	*p* value
Age, years, mean (SD)	24.3 (4.4)	21.3 (2.8)	24.5 (4.7)	0.15
Marital status, married (*n*, %)	10 (12.5)	0	2 (18.2)	0.45
Medical insurance status (*n*, %)				1.00
Medicaid	60 (75)	7 (77.8)	8 (72.7)	
Private	20 (25)	2 (22.2)	3 (27.3)	
Education (*n*, %)				
<HS diploma	14 (17.5)	1 (11.1)	3 (27.3)	0.18
HS diploma	19 (23.8)	5 (55.6)	4 (36.4)	
Some college	35 (43.8)	2 (22.2)	3 (27.3)	
College grad	7 (8.8)	1 (11.1)	1 (9.1)	
First prenatal BMI, mean (SD)	28.7 (7.8)	26.3 (4.5)	30.6 (11.4)	0.33
Parity, nulliparous (*n*, %)	33 (41.3)	7 (77.8)	8 (72.7)	0.03
Gestational weeks at enrollment, mean (SD)	11.4 (2.3)	10.9 (1.7)	10.8 (2.0)	0.61

**Table 2:** Summary of metabolic pathways showing significant enrichment for intergroup comparisons.

Preeclampsia vs. healthy control	Gestational hypertension vs. healthy control	Gestational hypertension vs. preeclampsia
Porphyrin metabolism	Fructose and mannose metabolism	Vitamin A metabolism
C21-steroid hormone biosynthesis	Aspartate and asparagine metabolism	Purine metabolism
Vitamin A metabolism	Glycine, serine, alanine, and threonine metabolism	
Arachidonic acid metabolism	Arginine and proline metabolism	
	Urea cycle/amino group metabolism	

## Data Availability

The data used to support the findings of this study are available from the corresponding author upon request.

## Supplementary Materials

Supplementary Materials Supplemental Figure 1: combined PE and gHTN compared to healthy full term. (A) Type 1 Manhattan plot, -log_10_*p* vs. mass-to-charge ratio. 348 *m*/*z* features were found significant at *p* value 0.05. No metabolites were significant by false discovery rate (FDR) *q* value 0.20. Red dots represent those features downregulated in gestational hypertension (HDP), and the blue dots represent features that were upregulated in HDP. (B) Type 2 Manhattan plot, -log_10_*p* vs. retention time. (C) 2-way hierarchical cluster analysis. HDP is represented in red and healthy full term in green across the *x*-axis; significant features are clustered on the *y*-axis; there is separation between the two groups of women. (D) Principal component analysis.
