# Supplementary material for: Early Pregnancy Serum Metabolite Profiles Associated with Hypertensive Disorders of Pregnancy in African American Women: A Pilot Study
Source: J Pregnancy. 2020 Feb 19;2020:1515321. doi: 10.1155/2020/1515321 (PMC7049834; doi:10.1155/2020/1515321)

Supplemental File

**Supplemental Figure 1: Combined PE and gHTN compared to Healthy Fullterm**

Supplemental Figure . A) Type 1 Manhattan plot, -log10p vs mass-to-charge ratio. 348 m/z features were found significant at p-value 0.05. ; No metabolites were significant by false discovery rate (FDR) q-value 0.20. Red dots represent those features down-regulated in gestational hypertension (HDP) and the blue dots represent features that were up-regulated in HDP. B) Type 2 Manhattan plot, -log10p vs retention time. C) 2-way Hierarchical Cluster Analysis, HDP is represented in red and healthy fullterm in green across the x-axis, significant features are clustered on the y-axis, there is separation between the two groups of women; D) Principal components analysis


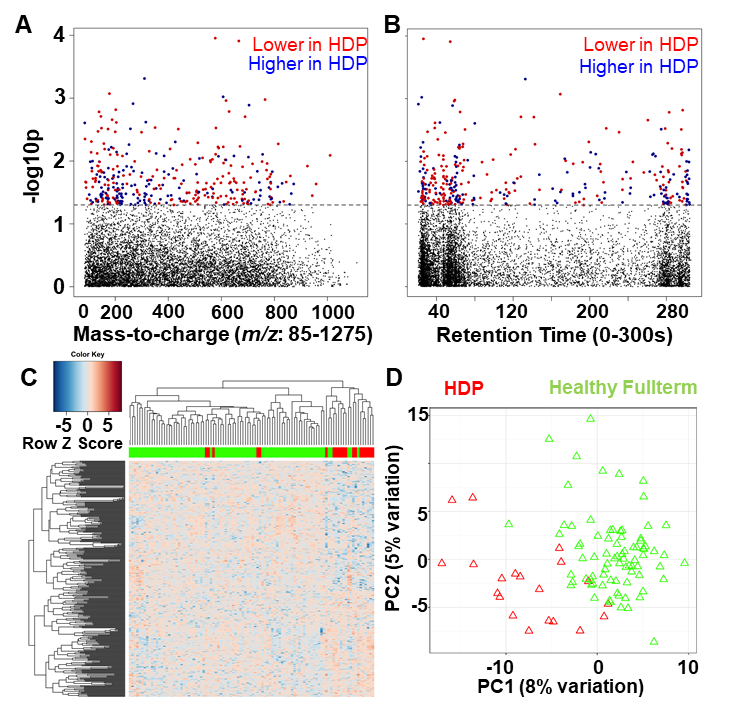

Supplement: Supplementary Materials — Supplemental Figure 1: combined PE and gHTN compared to healthy full term. (A) Type 1 Manhattan plot, -log10p vs. mass-to-charge ratio. 348 m/z features were found significant at p value 0.05. No metabolites were significant by false discovery rate (FDR) q value 0.20. Red dots represent those features downregulated in gestational hypertension (HDP), and the blue dots represent features that were upregulated in HDP. (B) Type 2 Manhattan plot, -log10p vs. retention time. (C) 2-way hierarchical cluster analysis. HDP is represented in red and healthy full term in green across the x-axis; significant features are clustered on the y-axis; there is separation between the two groups of women. (D) Principal component analysis. [file 1515321.f1.docx]
